# Duration of Keeping an Exercise Habit and Mental Illness and Life Attitude among University Students

**DOI:** 10.3390/ijerph191811669

**Published:** 2022-09-16

**Authors:** Lingfeng Kong, Yufei Cui, Qiang Gong

**Affiliations:** 1 Department of Physical Education, Hohai University, 1 Xikang Road, Nanjing 210098, China; 2 Department of Physical Education, Huaiyin Institute of Technology, Huaian 223003, China; 3 Department of Medicine and Science in Sports and Exercise, Tohoku University Graduate School of Medicine, Sendai 980-8575, Japan

**Keywords:** exercise habit, mental illness, young adults, cross-sectional study

## Abstract

Physical exercise has beneficial effects on human health, and several studies have examined the association between exercise and mental health. However, most of these studies focused on exercise intensity, duration, or frequency. Evidence for the association between the duration of maintaining an exercise habit and mental illness is lacking, especially in young adulthood—a crucial period that bridges adolescence and adulthood. This study investigated the association between the duration of keeping an exercise habit and mental illness and life attitude among Chinese university students. A total of 11,392 university students participated in this study (6866 men and 4526 women). The duration of keeping an exercise habit was evaluated using a questionnaire with relevant questions. Exercise habit was defined as exercising for longer than 30 min per session and more than twice a week. Mental illness consisted of two elements: depressive symptoms—assessed using the Zung Self-rating Depression Scale, and anxiety symptoms—assessed using the seven-item Generalized Anxiety Disorder scale. Life attitude was assessed using a questionnaire with relevant questions. Multivariate logistic regression analysis examined the adjusted association between the duration of keeping an exercise habit and mental illness and life attitude. In the final adjusted model, compared to the no exercise category, the odds ratios and 95% confidence intervals (CIs) of depressive symptoms were 0.80 (0.70, 0.91) for those with an exercise habit of 1–4 months, and 0.72 (0.63, 0.83) for those with one of >4 months (*p* for trend <0.001). Additionally, when compared to participants with no exercise habit, the adjusted odds ratios (95% CIs) for anxiety symptoms were 1.01 (0.90, 1.14) for those with a habit of 1–4 months and 0.78 (0.69, 0.88) for those with one of >4 months (*p* for trend =0.001). A longer duration of keeping an exercise habit was also significantly associated with positive life attitudes. Our results showed that a long duration of keeping an exercise habit was significantly associated with a lower prevalence of mental illness among university students. Further, maintaining a more prolonged exercise habit may benefit individuals’ mental health in young adulthood.

## 1. Introduction

The incidence and burden of mental illness have increased dramatically in recent years, making it a major health concern [1]. Mental illnesses are health conditions involving emotional changes, such as depressive and anxiety symptoms [2]. These symptoms are reportedly related to many negative health outcomes such as ischemic heart disease [3], physical disability [4], and suicide risk [5]. It has been speculated that mental health disorders are a leading cause of disability in the young population [6]. Furthermore, young adults at increased risk of mental disorders have severe social and occupational dysfunction [7]. A higher risk of developing depressive symptoms has been reported among university students in comparison to the general population [8]. Additionally, life attitudes are considered a complete process of psychological response, including needs, desires, psychological tendencies, and emotions present in the mind and expressed through behavior [9]. An optimistic attitude toward life affects subjective well-being and personal satisfaction, results in higher life satisfaction, and significantly reduces psychological problems [10]. Young adulthood is an important period that connects adolescence and adulthood. Mental health and life attitude during this period may predict future health status. Therefore, it is important to maintain good mental health and life attitude during this period.

There are many ways to improve mental health and prevent mental illness. Among these, exercise has been identified as an effective method. Exercise reduces inflammation [11], produces beneficial effects on neuroprotection [12], and provides a distraction from negative thoughts and ruminations [13]. All these effects can contribute to better mental health. Studies have examined the association between physical activity and mental health. However, based on literature, the definition of physical exercise is “physical activity that is planned, structured, repetitive, and purposeful in the sense that improvement or maintenance of one or more components of physical fitness is an objective” [14] that is contained in physical activity but differs from the definition of physical activity of “any bodily movement produced by skeletal muscles that requires energy expenditure” [15]. Physical activity in previous studies included not only physical exercise, but also physical activity in work and housework. In contrast, other studies that investigated the association between physical exercise and mental health mainly focused on specific exercise elements, such as exercise type (e.g., aerobic and strength) [16,17], exercise frequency [18,19], and exercise intensity [20,21]. Although some randomized trial studies used intervention duration as a conditional variable when examining the effects of exercise on mental health, participants in most of these studies were patients or older adults. Given that the effects of chronic and acute exercise on mental health may differ, the time taken to maintain an exercise habit may also be a crucial element for physical exercise. However, only one study has examined the association between exercise habit duration and depressive symptoms in 1429 older Chinese women aged 60 years and over. This indicated that a longer exercise habit duration (over one year) is associated with a lower risk of developing depressive symptoms [22]. These previous studies reveal a lack of evidence on this association in the young adult population. Nevertheless, the effects of exercise on mental illness may differ between young and older people. Additionally, to the best of our knowledge, no results have been found on the relationship between exercise and life attitudes. Therefore, we designed a cross-sectional study to investigate whether the duration of exercise habit is associated with mental illness and life attitude among young adults. In this study, we also considered some factors as confounders that may mediate or influence the association between exercise habits and mental illness or life attitudes, such as BMI, daily physical activity, smoking, and drinking status. These factors have been shown to be related to mental status [23,24,25]. Furthermore, grade was used instead of age in the study because students have different lifestyles in each grade, which may lead to different exercise habits or mental statuses.

## 2. Materials and Methods

### 2.1. Participants

We recruited participants through an annual physical health examination of university students at the Huaiyin Institute of Technology, Jiangsu, China, in 2018. This examination was conducted on all university students. We conducted a questionnaire survey before the examination under the guidance of the instructors. All participants voluntarily participated in the study, and written consent was obtained from them. This study was approved by the Ethics Committee of the Huaiyin Institute of Technology. A total of 12,580 students agreed to participate in the study and provided consent for data analysis. Participants with unavailable data on the Zung Self-rating Depression Scale (SDS), Generalized Anxiety Disorder (GAD-7) scale, and duration of keeping an exercise habit (*n* = 797) were excluded. Additionally, 391 students with data unavailable for body mass index (BMI), race, grade, living status, living expenses, physical activity, and smoking and drinking status were excluded. Thus, we recruited 11,392 participants (6866 men and 4526 women).

### 2.2. Assessment of Duration of Keeping an Exercise Habit

The duration of keeping an exercise habit was assessed using the question, “Do you have a habit of exercising for longer than 30 min per session and more than twice a week?” [26,27] and required a “yes” or “no” response. Students who responded “yes” to the question were then asked to indicate the duration of their exercise habit. The reported duration of keeping an exercise habit further categorized participants into three groups: those with no exercise habit, those with an exercise habit of 1–4 months, and those with an exercise habit of >4 months.

### 2.3. Assessment of Mental Illness

#### 2.3.1. Depressive Symptoms

Depressive symptoms were assessed using the Chinese version of the Zung SDS. The Zung SDS is commonly used to screen for depression in large patient populations to estimate its severity. The SDS is a 20-question self-report instrument with good internal consistency and validity, and it encompasses most Diagnostic and Statistical Manual-IV (DSM-IV) criteria for depression [28,29]. The SDS is the primary discriminating variable for distinguishing patients with depression from those with no depression. SDS index scores can range from 20 to 80. In this study, a cut-off score of 40 was used to define depressive symptoms and a cut-off score of 45 for sensitivity analysis [30]. The test score reliability coefficient for SDS was 0.80 using Cronbach’s alpha.

#### 2.3.2. Anxiety Symptoms

Anxiety symptoms were assessed using the GAD-7, a self-report questionnaire used for screening for anxiety symptoms. It consists of seven items to assess how long the respondents were troubled by seven problems. Participants were asked to score their anxiety-related issues during the preceding two weeks using a 4-point scale (0 = not at all; 3 = almost every day). The total scores can range from 0 to 21, with higher scores indicating increased severity of symptoms. The GAD-7 has been proven to be a reliable tool for measuring anxiety. In this study, cases with anxiety symptoms were defined as having a total score of GAD-7 ≥ 5 [31], and a GAD-7 score ≥ 8 was used for sensitivity analysis [32]. The test score reliability coefficient for GAD-7 was 0.94 using Cronbach’s alpha.

### 2.4. Assessment of Life Attitude

Life attitude was assessed using two questions: (1) “Do you think your life is meaningful?” and (2) “Do you have a purpose in your life?” The possible answers to these two questions were “yes” and “no”. The test score reliability coefficient for life attitude was 0.69 using Cronbach’s alpha.

### 2.5. Confounding Factors

As mentioned above, influential factors exist in the association between exercise habits, mental status, and life attitude. These factors are defined as follows. Body weight and height were measured, and BMI was calculated as weight (kg)/height (m)^2^. Daily physical activity was estimated using the International Physical Activity Questionnaire (IPAQ) [33]. Sex, grade, race, living expenses, living status, smoking status, and alcohol consumption were assessed using a self-reported questionnaire. The college grades were categorized into first-year students, sophomores, juniors, and seniors. Living expenses were classified into three categories: low (≤1000 yuan/month), medium (1001–1500 yuan/month), and high (>1500 yuan/month). The participants were classified into Han and minority groups, living status was classified as dormitory or others, and smoking status was classified as smoker and nonsmoker. Drinking status was categorized as nondrinker or drinking more than once per week.

### 2.6. Statistical Analyses

The differences in the variables among the duration of keeping exercise habit categories in participant characteristics were examined using the t-test or analysis of variance for continuous variables or the x^2^ test for categorical variables. Multivariate logistic regression analysis was used to examine the adjusted associations between duration of exercise habit and mental illness and life attitude. The duration of keeping an exercise habit was used as an independent variable, and mental illness and life attitude were dependent variables. Model 1 was adjusted for sex, grade, BMI, and race, and Model 2 was adapted for the items in Model 1 plus living expenses, physical activity, living status, smoking status, and drinking status. All statistical analyses were performed using the SPSS Windows software version 24.0. The results were presented as odds ratios with 95% confidence intervals (CIs), and a *p*-value less than 0.05 was considered statistically significant.

## 3. Results

Participant characteristics according to the duration of keeping an exercise habit are shown in Table 1. With the increasing duration of exercise habits, the proportions of first-year students, nonsmokers, and nondrinkers decreased significantly (*p* for trend <0.001, <0.001, and <0.001, respectively). In contrast, the proportions of men, seniors, and people with high living expenses increased significantly (*p* for trend <0.001, <0.001, and =0.010, respectively). Meanwhile, a longer duration of keeping an exercise habit was significantly associated with a higher BMI, higher physical activity, and lower SDS and GAD-7 scores.

Table 2 presents the adjusted association between the duration of keeping an exercise habit and depressive symptoms. In the final adjusted model of SDS cut-off point 40 (Model 2), when compared with participants with no exercise habit, the adjusted odds ratios (95% CIs) of depressive symptoms were 0.80 (0.70, 0.91) for those with an exercise habit of 1–4 months, and 0.72 (0.63, 0.83) for those with an exercise habit of >4 months (*p* for trend <0.001). The sensitivity analysis also found the same significant relationship (SDS cutoff point: 45).

The adjusted association between the duration of keeping an exercise habit and anxiety symptoms is presented in Table 3. In the final adjusted model of GAD-7 cut-off point 5 (Model 2), when compared with participants with no exercise habit, the adjusted odds ratios (95% Cis) of anxiety symptoms were 1.01 (0.90, 1.14) for those with an exercise habit of 1–4 months and 0.78 (0.69, 0.88) for those with an exercise habit of >4 months (*p* for trend =0.001). The same significant relationship was also found in the sensitivity analysis (GAD-7 cutoff point 8).

The adjusted association between the duration of keeping an exercise habit and life attitude is presented in Table 4. In the final adjusted model of life is meaningless (Model 2), when compared to participants with no exercise habit, the adjusted odds ratios (95% Cis) of life is meaningless were 0.70 (0.59, 0.84) for those with an exercise habit of 1–4 months, and 0.62 (0.51, 0.76) for those with an exercise habit >4 months (*p* for trend <0.001). In the final adjusted model of no purpose in life, when compared with participants with no exercise habit, the adjusted odds ratios (95% Cis) of life is meaningless were 0.69 (0.58, 0.82) for those with an exercise habit of 1–4 months, and 0.48 (0.39, 0.59) for those with an exercise habit >4 months (*p* for trend <0.001).

## 4. Discussion

This study investigated the association between the duration of keeping an exercise habit and mental illness and life attitude among university students. The results suggest that a longer duration of keeping an exercise habit is associated with a lower risk of mental illness, including depressive and anxiety symptoms, and a positive life attitude. In addition, these associations did not change after adjusting for several potential confounding factors. To the best of our knowledge, this is the first study to examine the association between the duration of keeping an exercise habit and mental illness and life attitude in a young adult population. Our findings provide crucial information on preventive medicine and health education.

The mean SDS score of our participants was 35.6 (SD 7.1). This result is consistent with a Chinese study from 2019, which reported a mean score of 37.1 (SD 8.0) among university students [34]. However, it is a little lower than another Chinese study and a Russian study, which showed mean SDS scores for university students of 44.4 (SD 9.9) and 47.8 (SD 10), respectively [35,36]. In contrast, the mean GAD-7 scores reported in previous studies (including a Chinese, an Ethiopian, and an American study) were more than 6 in university students [37,38,39]. These scores were higher than our study’s (mean GAD-7 score = 3.85).

In light of this study’s research objectives and contents, we found several pieces of literature relevant to our study from significant databases. An intervention study of 185 university students confirmed that regular engagement in low- to moderate-intensity aerobic exercises for 6 weeks effectively alleviates subclinical depressive symptoms and perceived stress [40]. Another intervention study on 30 university students showed that a 1-h-long Tai Chi workout twice a week for 3 months positively affected self-assessed mental health [41]. These two previous studies examined whether maintaining a period of exercise can affect the mental health of university students. However, the design of these studies differed from that of ours, as they observed exercise habits for 6 weeks and 3 months, respectively, which may have only examined the short-term effects of exercise on mental health. Conversely, one cross-sectional study of 472 university students examined the influence of physical exercise on college students’ mental health [42]. Nevertheless, the duration of keeping an exercise habit was not used as a separate variable in this study; instead, it was combined with other exercise elements (frequency and intensity) to form a new variable (amount of exercise). The results of that study showed that physical exercise had a positive effect on the mental health of college students, which is partially consistent with our findings. In addition, a cross-sectional study of 1429 older Chinese women indicated that a long duration of exercise habit is associated with a lower risk of depressive symptoms [22]. Although the age of the participants differed from that of our participants, this result is consistent with our findings, and we extended their findings to a young adult population. All these previous studies directly or indirectly confirmed the association between the duration of keeping an exercise habit and mental illness. The main differences between these studies and our study are as follows: First, we used different scales to evaluate mental illness and the duration of keeping an exercise habit. Second, the participants were from different countries and of different ages. Third, the design and objectives of these studies were different. Our study mainly focused on the association between the duration of keeping an exercise habit and mental illness. Fourth, the association between exercise habits and life attitude was not considered and examined in those previous studies. The strength of our study is that we had a larger sample size than previous studies and used two cut-off values for SDS and GAD-7 to make the results more credible. Thus, our study strengthens evidence of the association between physical exercise and mental health. Moreover, a review article that investigated physical activity habits of university students concluded that there are gender differences in physical activity and obstacles to the practice of physical activity in university students [43]. Although gender differences and obstacles to physical activity were not examined in this study, this review article highlights the need to consider these factors in future studies.

Some mechanisms may explain why a long duration of keeping an exercise habit is associated with better mental health and life attitude. Studies have shown that exercise benefits neuronal function, including neuroprotection [12,44]. Voluntary exercise significantly increases brain-derived neurotrophic factor (BDNF) in the hippocampus [45]. The regulation of BDNF is observed in the same subfields of the hippocampus as shown for antidepressant treatment. Furthermore, exercise has been shown to increase the expression and levels of growth factors such as IGF-1 [46], which have antidepressant effects. Lastly, the long-term effects of chronic physical exercise appear to result in different responses and adaptations than those observed following acute exercise [47]. Antioxidant effects have been observed in people who have exercised for an extended period [48]. Oxidative stress has been associated with depressive symptoms [49]. Thus, a long-term exercise habit may mitigate mental illness and prevent mental illness deterioration.

Notably, the present study has certain limitations. First, because this was a cross-sectional study, it was challenging to draw conclusions regarding causality. Second, self-reported questionnaires were used in this study; thus, recall bias might exist, and the actual lifestyle of the participants might not have been reflected accurately. Third, the data in this study were collected from university students; thus, the results may not represent the total population. Fourth, because of the limited survey time, we evaluated life attitudes using only two questions, not a validated questionnaire. Thus, the complete life attitude of the participants may not be represented. Finally, although we adjusted for several potential confounding variables, we could not exclude the possibility that other covariates may have influenced the association between the duration of keeping an exercise habit and mental illness.

## 5. Conclusions

In conclusion, the present study found that a longer duration of keeping an exercise habit was associated with a lower risk of mental illness and a positive life attitude among university students. Therefore, our findings imply that maintaining an exercise habit is recommended to prevent mental illness problems among university students. Further prospective epidemiological studies or randomized studies are required to confirm these findings and establish causality.

## Figures and Tables

**Table 1:** Participant characteristics according to categories of duration of keeping an exercise habit.

	Duration of Keeping an Exercise Habit			
	None	1–4 Months	>4 Months	*p* for Trend ^a^
n	8482	1465	1445	
Sex (man; %)	57.0	60.8	78.9	<0.001
BMI ^b^ (kg/m^2^)	21.3 (21.2, 21.4) ^c^	21.8 (21.7, 22.0)	21.5 (21.4, 21.7)	0.009
Grade (%)				
First-year students	31.2	27.0	26.0	<0.001
Sophomore	29.7	33.3	28.1	0.890
Junior	27.1	27.4	27.0	0.955
Senior	12.0	12.2	18.9	<0.001
Living expenses (%)				
Low	37.8	38.4	36.7	0.581
Medium	52.5	51.0	51.4	0.311
High	9.7	10.6	11.8	0.010
Minority race (%)	4.4	4.8	5.6	0.050
Living status (Dormitory; %)	99.1	99.2	99.0	0.873
Nonsmoker (%)	95.5	93.0	91.2	<0.001
Nondrinker	81.3	77.1	68.4	<0.001
PA (METs hour/week)	1.53 (1.53, 1.54) ^d^	1.68 (1.66, 1.70)	1.78 (1.76, 1.80)	<0.001
SDS score	35.9 (35.7, 36.1)	35.0 (34.6, 35.4)	34.2 (33.8, 34.5)	<0.001
GAD-7 score	3.94 (3.87, 4.02)	3.88 (3.70, 4.06)	3.29 (3.10, 3.47)	<0.001

^a^ Obtained using analysis of variance for continuous variables and x^2^ test for proportional variables; ^b^ BMI: body mass index; PA: physical activity; SDS: self-rating depression scale; GAD-7: general anxiety disorder; ^c^ Mean; 95% CI in parentheses (all such values). ^d^ Variables were log-transformed for normal distribution.

**Table 2:** Adjusted association for depressive symptoms according to categories of duration of keeping an exercise habit.

	Duration of Keeping an Exercise Habit			
	None	1–4 Months	>4 Months	*p* for Trend ^a^
All subjects (n)	8482	1465	1445	
Depressive symptoms (SDS ≥ 40; n)	2456	362	323	
Crude	1.00	0.81 (0.71, 0.92) ^b^	0.71 (0.62, 0.81)	<0.001
Model 1 ^c^	1.00	0.80 (0.70, 0.91)	0.72 (0.63, 0.83)	<0.001
Model 2 ^d^	1.00	0.81 (0.71, 0.92)	0.74 (0.64, 0.85)	<0.001
Depressive symptoms (SDS ≥ 45; n)	1065	143	151	
Crude	1.00	0.75 (0.63, 0.91)	0.81 (0.68, 0.97)	0.002
Model 1 ^c^	1.00	0.75 (0.62, 0.90)	0.80 (0.67, 0.96)	0.001
Model 2 ^d^	1.00	0.75 (0.62, 0.90)	0.80 (0.66, 0.96)	0.002

^a^ Obtained by multiple logistic regression analysis; ^b^ Adjusted OR; 95% CI in parentheses (all such values); ^c^ Adjusted for grade, sex, race, and BMI; ^d^ Adjusted for Model 1 plus living expenses, physical activity, living status, and smoking and drinking habits.

**Table 3:** Adjusted association for anxiety symptoms according to categories of duration of keeping an exercise habit.

	Duration of Keeping an Exercise Habit			
	None	1–4 Months	>4 Months	*p* for Trend ^a^
All subjects (n)	8482	1465	1445	
Anxiety symptoms (GAD7 ≥ 5; n)	3713	632	526	
Crude	1.00	0.97 (0.87, 0.09) ^b^	0.74 (0.66, 0.83)	<0.001
Model 1 ^c^	1.00	1.01 (0.90, 1.13)	0.78 (0.69, 0.88)	<0.001
Model 2 ^d^	1.00	1.01 (0.90, 1.14)	0.78 (0.69, 0.88)	0.001
Anxiety symptoms (GAD7 ≥ 8; n)	1078	173	137	
Crude	1.00	0.92 (0.78, 1.09)	0.72 (0.60, 0.87)	0.001
Model 1 ^c^	1.00	0.94 (0.79, 1.11)	0.78 (0.64, 0.94)	0.010
Model 2 ^d^	1.00	0.94 (0.79, 1.11)	0.78 (0.64, 0.94)	0.012

^a^ Obtained by multiple logistic regression analysis; ^b^ Adjusted OR; 95% CI in parentheses (all such values); ^c^ Adjusted for grade, sex, race, and BMI; ^d^ Adjusted for Model 1 plus living expenses, physical activity, living status, and smoking and drinking habits.

**Table 4:** Adjusted association for life attitude according to categories of duration of keeping an exercise habit.

	Duration of Keeping an Exercise Habit			
	None	1–4 Month	>4 Months	*p* for Trend ^a^
All subjects (n)	8482	1465	1445	
Life is meaningless	1243	151	120	
Crude	1.00	0.67 (0.56, 0.80) ^b^	0.53 (0.43, 0.64)	<0.001
Model 1 ^c^	1.00	0.65 (0.54, 0.78)	0.54 (0.44, 0.66)	<0.001
Model 2 ^d^	1.00	0.70 (0.59, 0.84)	0.62 (0.51, 0.76)	<0.001
No purpose in life	1409	172	115	
Crude	1.00	0.67 (0.56, 0.79)	0.43 (0.36, 0.53)	<0.001
Model 1 ^c^	1.00	0.65 (0.55, 0.77)	0.43 (0.35, 0.52)	<0.001
Model 2 ^d^	1.00	0.69 (0.58, 0.82)	0.48 (0.39, 0.59)	<0.001

^a^ Obtained by multiple logistic regression analysis; ^b^ Adjusted OR; 95% CI in parentheses (all such values); ^c^ Adjusted for grade, sex, race, and BMI; ^d^ Adjusted for Model 1 plus living expenses, physical activity, living status, and smoking and drinking habits.

## Data Availability

The datasets used and analyzed during the current study are available from the corresponding author on reasonable request.
